# Nutritional Criminology: Why the Emerging Research on Ultra-Processed Food Matters to Health and Justice

**DOI:** 10.3390/ijerph21020120

**Published:** 2024-01-23

**Authors:** Susan L. Prescott, Alan C. Logan, Christopher R. D’Adamo, Kathleen F. Holton, Christopher A. Lowry, John Marks, Rob Moodie, Blake Poland

**Affiliations:** 1 School of Medicine, University of Western Australia, Perth, WA 6009, Australia; susan.prescott@telethonkids.org.au; 2 Nova Institute for Health, Baltimore, MD 21231, USA; cdadamo@som.umaryland.edu; 3 Department of Family and Community Medicine, School of Medicine, University of Maryland, Baltimore, MD 21201, USA; 4 The ORIGINS Project, Telethon Kids Institute, Perth, WA 6009, Australia; 5 Departments of Health Studies and Neuroscience, Center for Neuroscience and Behavior, American University, Washington, DC 20016, USA; holton@american.edu; 6 Department of Psychology and Neuroscience, Center for Neuroscience and Center for Microbial Exploration, University of Colorado Boulder, Boulder, CO 80309, USA; christopher.lowry@colorado.edu; 7 Department of Criminal Justice, Louisiana State University of Alexandria, Alexandria, LA 71302, USA; jmarks@lsua.edu; 8 School of Population and Global Health (MSPGH), University of Melbourne, Melbourne, VIC 3052, Australia; r.moodie@unimelb.edu.au; 9 Dalla Lana School of Public Health, University of Toronto, Toronto, ON M5R 0A3, Canada; blake.poland@utoronto.ca

**Keywords:** aggression, behavior, diet, food crime, mental health, microbiome, nutrition, ultra-processed foods

## Abstract

There is mounting concern over the potential harms associated with ultra-processed foods, including poor mental health and antisocial behavior. Cutting-edge research provides an enhanced understanding of biophysiological mechanisms, including microbiome pathways, and invites a historical reexamination of earlier work that investigated the relationship between nutrition and criminal behavior. Here, in this perspective article, we explore how this emergent research casts new light and greater significance on previous key observations. Despite expanding interest in the field dubbed ‘nutritional psychiatry’, there has been relatively little attention paid to its relevancy within criminology and the criminal justice system. Since public health practitioners, allied mental health professionals, and policymakers play key roles throughout criminal justice systems, a holistic perspective on both historical and emergent research is critical. While there are many questions to be resolved, the available evidence suggests that nutrition might be an underappreciated factor in prevention and treatment along the criminal justice spectrum. The intersection of nutrition and biopsychosocial health requires transdisciplinary discussions of power structures, industry influence, and marketing issues associated with widespread food and social inequalities. Some of these discussions are already occurring under the banner of ‘food crime’. Given the vast societal implications, it is our contention that the subject of nutrition in the multidisciplinary field of criminology—referred to here as nutritional criminology—deserves increased scrutiny. Through combining historical findings and cutting-edge research, we aim to increase awareness of this topic among the broad readership of the journal, with the hopes of generating new hypotheses and collaborations.

## 1. Introduction

Over the last decade, the wide-ranging consequences of an international food supply rich in ultra-processed foods have entered scientific and public conversations [1,2,3]. Ultra-processed foods are generally recognized as being high in refined sugar and/or industrial fat and/or sodium and/or inclusive of emulsifiers, plant isolates, extruded meat remnants, flavor enhancers, colors, and other synthetic additives. Depending on their formulation, they can be dense in calories, low in nutrients, and low in fiber. At the same time, major media outlets have highlighted the potential harms of ultra-processed foods [4,5,6], and a growing number of Op-Ed pieces are calling for serious policy reforms aimed at curbing their widespread availability [7,8,9]. Calls to tax, label, reduce advertising for (particularly to children), and even ban ultra-processed foods are bolstered by a growing body of research connecting highly processed foods to increased mortality [10,11,12,13,14,15], as well as the risk of a variety of noncommunicable diseases [16,17,18].

**Figure 1:**
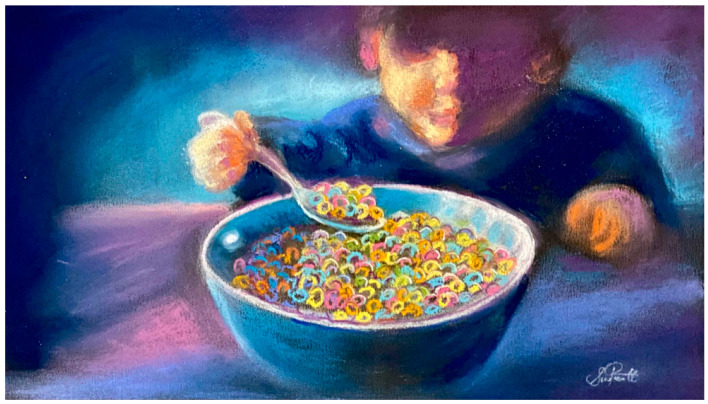
The rise in ultra-processed foods in the 20th century is a key factor implicated in the rise in the pandemic of noncommunicable physical and mental diseases through immune and metabolic effects beginning early in life. Growing evidence linking ultra-processed foods to various neuropsychiatric outcomes and antisocial and/or aggressive behavior has major implications for the criminal justice system and society at large (artwork copyright, author S.L.P.).

Mostly, recent mainstream media attention and policy discussions have focused on ultra-processed foods as a potential causative factor in obesity, cardiovascular disease, and type II diabetes. Part of this discourse has included emerging evidence pointing toward the addictive potential of ultra-processed foods [19,20,21]. Less attention has been paid to the growing relevance the nutrition sciences have to the behavioral sciences—research linking ultra-processed foods to mental disorders and various neuropsychiatric outcomes, including depression, anxiety [22,23,24,25,26,27,28,29,30], and antisocial and/or aggressive behavior [31,32,33,34,35,36,37] (Figure 1). The extent to which industry is responsible for promoting noncommunicable diseases, including mental disorders and addiction to ultra-processed food-like products, is described by criminal justice researchers under the banner of ‘food crime’ [38,39].

Here, in our perspective article, we draw from a variety of sources, such as PubMed, Google Scholar, Google Books, and newspaper archives. We discuss this emerging research in a historical context, focusing on a particular time when there was some enthusiasm for the potential role of nutrition in criminology, the 1980s. These historical findings are then paired with contemporary research; ultra-processed food consumption has recently been associated with low-grade systemic inflammation [40,41] and appears to influence systemic metabolites and energy uptake via changes to the gut microbiome [42,43,44]. These are mechanistic topics to be discussed in more detail below. It is our contention that emergent research, including an enhanced understanding of biophysiological mechanisms [45,46], has placed some early theorists on the right side of history. Concepts such as ‘nutritional psychiatry’ are steeped in history, and to the extent that such history is ignored, it acts as a barrier to the dissemination of critical knowledge, maintains silos, prevents researchers from seeing the relevancy of findings to their own work, and is, on its face, ethically problematic [47,48]. From the outset, we underscore that food processing technology has been of vital importance to human health and wellbeing for millennia. Our context here is ultra-processed products that are increasingly connected to harm [49]. Our objective is to increase awareness of this topic among the broad, transdisciplinary readership of the journal, in the hopes that it will generate new hypotheses and collaborations. While there are many questions to be resolved, we argue that the available evidence suggests that dietary components are an underappreciated factor in prevention and treatment throughout the criminal justice spectrum.

## 2. Diet and Antisocial Behavior—Perspectives across the 20th Century

The notion that diets influence brain and behavior is not a new idea. Writing in the *Journal of the American Medical Association* in 1899, psychiatrist Henry C. Eyman stated that a “proper diet is our most powerful agent” in the treatment of depression [50]. In 1954, Drs. George Watson and associate Andrew L. Comrey studied the potential of nutritional supplements in treating mental disorders. In a single-blind controlled study (*n* = 34) published in the *Journal of Psychology*, they found that an oral vitamin–mineral supplement (with some added ingredients such as alfalfa, watercress, seaweed, parsley, etc.) could improve symptoms in adults with depression and anxiety [51]. Although Watson and Comrey’s report went largely unnoticed in scientific circles, the findings were disseminated to the public via syndicated newspaper articles [52]. Among the readers of Watson and Comrey’s paper was actress and fashion designer Gloria Swanson; she became a strong proponent of healthy dietary practices as part of the prevention of juvenile delinquency. Swanson would quote from Watson and Comrey’s papers [53] while publicly claiming that “*I can’t believe juvenile delinquency doesn’t come from the lack of proper nutrition. If the body can become sick from this, then why not the mind?*” [54]. The media noted that Swanson’s specific “war is with processed foods” [54]. Swanson’s campaign to address juvenile delinquency through reducing processed foods and introducing healthy alternatives was noted in nutrition journals at the time [55]. When Swanson was campaigning on her suspicions that highly processed foods were contributing to juvenile delinquency, she blamed “adult delinquents” and “lawmakers and special interest groups with selfish interests” who allowed for policies and practices that were making highly processed, additive-containing foods the norm rather than the exception [54].

Watson’s popular press book, ‘*Nutrition and Your Mind’*, was published in 1972 [56]. Introduced with much fanfare and considerable attention from the popular press, the book generated magazine cover headlines. For example, Cosmopolitan magazine’s cover exclaimed “Can diet banish depression and cure emotional ills? It already has!” [57], while McCall’s magazine cover read “A startling new theory: Diet—not psychiatry—can cure mental illness” [58]. The problem for psychiatrists and other mental health professionals was that, despite the obvious public appeal, Watson’s book and Swanson’s “influencer” campaign were not girded by scientific support (mechanistic and/or epidemiological); Watson’s nutritional supplement studies from the 1950s [51,59,60], although interesting and perhaps worthy of greater attention, were not scientifically rigorous.

Watson was influential to the extent that he challenged prevailing Freudian psychoanalysis and queried whether, at least for some people, mental disorders were really ‘mental’ or a matter of biophysiology [61,62]. However, in 1977, the absence of science in the field of nutrition and behavior was illuminated by the United States Senate. In hearings intended to query the consequences of nutritional deficiencies and junk food on mental illness and juvenile delinquency, the Committee found an absence of evidence [63]. The Chair, Senator George McGovern, concluded that the topic of nutrition and behavior was poorly funded and warranted increased attention [64]. Despite the Senate’s acknowledgement of the lack of evidence, media headlines such as “Junk Food: What’s it doing to your mind?” [65] pushed ideas that would form the basis of the “Twinkie Defense” in criminal law [63].

One of the most influential witnesses at the Senate hearings in 1977 was Barbara J. Reed, an Ohio probation officer. Several years prior to the hearings, Reed had begun advocating for reducing processed food intake among probationers, with particular attention to avoiding foods with refined sugar and sugar-sweetened beverages. While anecdotal, Reed claimed the aforementioned dietary changes reduced antisocial behavior, aggression, and recidivism. Reed’s observations were coincident with a generalized concern about behavioral problems, especially in children, associated with sugar and various additives found in processed foods. Reed became a de facto spokesperson for the topic of nutrition and criminal behavior, with appearances on CBS News [66] and write-ups in *The Wall Street Journal* and a variety of magazines [67]. In their article on Reed, *The Washington Post* carefully noted that “the American Medical Association and many representatives of the medical establishment do not believe that diet plays a role in mental illness” [68]. The key word in the sentence is ‘believe’; at both ends of the spectrum, advocates and skeptics encapsulated by Reed and the American Medical Association, respectively, were operating on beliefs.

Reed acknowledged that diet was not an exclusive contributor to antisocial behavior and underscored that she wanted the topic to be researched by experienced scientists: “*I am not saying that food is to blame for every act of violence, but if crime can be reduced by even a small percentage, then I think it is something worth investigating*” [69]. One of the first scientists to answer Reed’s call was social scientist and criminologist Dr. Stephen J. Schoenthaler [63]. In the early 1980s, a chef in a Virginia juvenile detention facility working under the direction of a state nutritionist altered the institutional menu with the primary aim of lowering the overall content of refined sugar. Schoenthaler evaluated the frequency of antisocial behavior (as documented by institutional personnel) among juvenile subjects who had consumed the amended diet and compared them to juveniles who had arrived at the institution in the period before the dietary transition. The group on the amended diet was reported to have a 45 percent lower incidence of documented disciplinary actions [70]. According to Schoenthaler, the covert design was such that only the food preparation team was aware of the dietary changes, not the institutional personnel responsible for recording disciplinary actions. Perhaps it was for this reason that Schoenthaler originally described the study as double-blind and later conceded, under criticism, that it was an ‘open trial’ [71]. In any case, this was a preliminary effort involving 58 juveniles, of whom only 24 had experienced the dietary modification.

Schoenthaler’s initial results led to larger follow-up studies examining the removal of highly processed foods and the inclusion of healthier, less processed options at a number of different juvenile facilities, including the Tidewater detention facility in Virginia [72] and others in Los Angeles County, California [73], and Coosa Valley, Alabama [74]. From 1983 to 1985, Schoenthaler reported studies involving over 8000 juveniles with an average 47 percent reduction in infractions and indicators of antisocial behavior, ranging from overt violence, acts of theft, verbal aggression, and insubordination to facility employees. These offenses were documented by correctional personnel and compared to the periods prior to and after the dietary modifications [75,76].

While the intervention trials were reported in the media and initially by Schoenthaler as simple “sugar reduction”, the intervention was more specifically a swapping out of ultra-processed foods for less processed whole foods. For example, breakfast cereals with significant amounts of added sugar were replaced with those that were not presweetened, and tinned fruits with syrupy added sugars were either drained and rinsed or substituted with whole fruits. Sugar-sweetened beverages, soft drinks, imitation juices, and powered instant drink mixes were replaced with 100% juice derived from citrus and other fruit. High-sugar and high-fat snacks such as cookies and potato chips were replaced with fresh fruit, vegetables, cheeses, and whole-grain products. Whole-grain bread and brown rice replaced the refined white versions [63].

Even though Schoenthaler’s studies were a whole-of-diet approach, much of the mechanistic discourse focused on sugar because, at the time, it was thought that ‘reactive’ hypoglycemia was a driver of aggressive and antisocial behavior. Although tenuous, there was some research indicating that aggression could be provoked by moderate hypoglycemia and that violent offenders appeared to experience clinically meaningful hypoglycemia after sugar ingestion [77,78,79]. Hints that certain individuals are prone to hypoglycemic-induced aggression date back to the first half of the 20th century, including reports of irritability as a result of hypoglycemia, resolved with dietary changes reducing sugar and refined foods [80]. Joseph Wilder, a professor of neurology at New York Medical College, studied hypoglycemia and abnormal behavior for over a decade [81,82], concluding in the *Handbook of Correctional Psychology* that the irritability, emotional reactivity, and loss of central inhibition associated with hypoglycemia were linked to cases ranging from disorderly conduct to serious violence [83]. In a 1943 case report in *The Lancet*, physicians reported that a 20-year-old man who murdered his mother had abnormal electroencephalogram (EEG) readings when his blood glucose moderately dipped into the hypoglycemic range [84].

Schoenthaler’s work on dietary patterns in correctional facilities was subjected to significant criticism; California psychiatrist Gregory E. Gray opined that this area of research was “little more than pseudoscience”, going on to assert that nutritional psychiatry theories have public appeal because they may “relieve parents guilt about having been inadequate as parents” [85]. In November 1984, the American Medical Association, along with The Nutrition Foundation Inc., an industry conglomerate whose trustees included senior executives from Coca-Cola, General Mills, Dow Chemical, Kellogg’s, etc. [86], and the International Life Sciences Institute (ILSI) another industry collective that has since been reported to be a front group engaged in influence-peddling [87,88,89,90], convened a meeting on food and behavior in Arlington, Virginia. The ILSI, an outfit that maintains its own journal, *Nutrition Reviews* [91], quickly wrote up their conclusions of the meeting, disseminated them to the media, and published the take-home message in their journal: “*Evidence does not support diet as a significant etiologic factor in mediating such behaviors as hyperactivity and criminal behavior*” [92]. The American Dietetic Association (now called the Academy of Nutrition and Dietetics) followed in lockstep, quickly concluding that diet “is not an important determinant” of behavior [93]. Schoenthaler was at the meeting, but as noted in the *New York Times*, “[Schoenthaler] *had but a few minutes during a discussion period to answer the attacks of his critics*” [94].

Shortly after the AMA–ILSI–Nutrition Foundation Inc. symposium, psychiatrist Gray informed the *San Francisco Examiner* that nutritional psychiatry theories were problematic on their face because “*instead of placing responsibility for an individual’s criminal actions on himself, we’re shifting the blame to his food and providing no incentive for him to change his behavior*” [95]. This personal responsibility emphasis is the same one that positioned obesity as a lack of willpower or motivation [96] while ignoring the larger discussions of the total food environment [97]. Schoenthaler responded by stating that he was concerned about vulnerabilities and that social scientists should evaluate all potential predictors of antisocial behavior, be it nutrition, family dynamics, poverty (of which food insecurity is an important corollary), education, substance use, and so on [98]. In the conference proceedings published in ISLI’s *Nutrition Reviews*, Gray concluded that attempts to remove highly processed foods and replace them with minimally processed alternatives in correctional facilities, based on the work of Schoenthaler and others, were the “incorporation of food faddism into public policy” [99]. In 1980s medico-science culture, the sponsorship of a food and behavior conference by the Nutritional Foundation Inc. and the ILSI may not have seemed odd. However, recent evidence demonstrates the sway of the ultra-processed food industry when it sponsors academic conferences [100].

Schoenthaler subsequently abandoned studies involving whole-of-diet interventions and moved to placebo-controlled trials involving multi-vitamin–mineral formulas. In two randomized controlled trials involving the administration of a vitamin–mineral supplement to inmates in correctional facilities, Schoenthaler reported significant reductions in rule violations among subjects consuming the supplement [101,102]. These studies, since replicated internationally in diverse populations in confinement, indicate that basic multi-vitamin/mineral and modest omega-3 fatty acid supplementation might play a helpful role in reducing the frequency of disciplinary offenses [103], rule-breaking incidents [104], and aggression [105]. An ongoing study in Australia is evaluating the potential of omega-3 fatty acid supplementation to lower aggression among inmates within correctional facilities [106]. Previous research in Singapore has indicated that omega-3 fatty acid supplementation can reduce antisocial and aggressive behavior over and above regular treatment programs in young offender institutions [107] and may reduce post-release recidivism rates [108].

Links between low blood and brain tissue levels of omega-3 fatty acids and human aggression, homicide, and suicide by violent means have been discussed for over three decades [109,110,111]. Omega-3 fatty acids have been found to reduce aggression, antisocial behaviors, and self-harm in the healthy general population and populations with known neuropsychiatric disorders [112,113,114,115,116]. Inmates in correctional facilities may have lower blood levels of omega-3 than found in the wider population, with research further indicating that inmates with lower blood levels of omega-3 fatty acids are more aggressive than inmates with high Omega 3 Index scores [117].

In addition to Schoenthaler, other groups were examining the extent to which an elevated blood copper/zinc ratio might be contributing to aggression, lowered mood, cognitive difficulties, and criminality [118,119,120]. Such blood micronutrient ratios can be the result of poor diets lacking nutritional diversity and/or increased demands for zinc while under psychological/physiological stress [121,122]. While studies showing that isolated supplements might improve mood and reduce anger and irritability [123,124], are obviously relevant to the overall topic of nutrition and criminal behavior, our focus here is on ultra-processed foods; therefore, we will focus on studies involving food/dietary patterns and mental health and behavior. While dietary interventions directed at aggression, antisocial behavior, and criminal risk have largely been ignored since the 1980s, studies in the field of nutritional psychiatry, as described below, are of relevance. First, we will examine the contemporary research on sugar, aggression, and mental health.

## 3. Sugar Revisited

In the 1980s, the sugar industry, under the auspices of “Sugar Associates Inc” and “The Sugar Association”, funded Mark Wolraich and colleague Richard Milich to examine whether or not sugar administration influenced hyperactivity in children. They found that sugar-containing (vs. aspartame-containing) beverages did not influence post-consumption behavior [125,126]. In separate work funded by the aforementioned Nutrition Foundation Inc. and the ILSI, Wolraich and colleagues reported that separate 3-week blocks of consuming foods sweetened with sugar, aspartame, and saccharin on a retaining basis made no difference to behavior in children [127]. This highly influential study has been cited almost 500 times on Google Scholar, and Wolraich’s 1995 meta-analysis in the *Journal of the American Medical Association*, wherein his own studies are cited, concluded that sugar has no influence on childhood behavior or cognitive performance [127]. It has also been cited almost 500 times and continues to be used by the media as the primary reference to “debunk” any notions that sugar influences childhood behavior [128,129].

The problems with conducting a sugar vs. artificial sweetener beverage trial should have been obvious at the time. If the working hypothesis is that sugar can cause behavioral disruptions, why use bioactive chemicals now known (and certainly suspected then) to lead to changes in brain physiology and behavioral disturbances [130,131,132,133,134] as the placebo? At this point, no conclusions can be drawn from Wolraich’s industry-funded sugar vs. aspartame studies. The extent to which short- and long-term dietary sugar intake influences cognition, attention-deficit hyperactivity disorder, and externalizing behavior has not been resolved [135,136,137,138]. In a 2012 study involving the Boston school system, adolescents who drank more than five cans of soft drinks per week (almost 30% of the sample) were significantly more likely to have carried a weapon and to have been violent with peers, family members, and romantic partners. Frequent soft drink consumption was associated with a 9–15% point higher probability of engaging in aggressive actions, even after controlling for gender, age, race, body mass index, typical sleep patterns, tobacco use, alcohol use, and other factors [139]. In the British Cohort Study (*n* = 17,415), high amounts of confectionary consumption at age 10 were associated with increased odds of a violent crime conviction at age 35 [140]. Internationally, strong associations between sugar consumption, aggression, suicidal behavior, and risk taking have been observed [36,141,142,143]. It is possible that anxiety, irritability, and impulsivity are mediators in the dietary sugar–aggression associations [144,145].

Of course, these studies do not establish a causal relationship between sugar consumption and violence. Indeed, it might be the case that higher sugar consumption is a subconscious attempt to ‘self-medicate’ into enhanced self-control, as was originally theorized in the reactive hypoglycemia concept. Consider recent human studies showing that blood glucose levels predict performance on self-control tasks, acute consumption of glucose drinks attenuates self-control impairments [146,147], carbohydrate-rich/protein-poor food consumption stabilizes mood and attenuates stress reactivity in stress-prone individuals under experimental stress [148,149], and that glucose administration, in the acute phase, reduces aggression associated with experimentally manipulated social rejection [150]. These studies suggest that acute sugar administration can, at least for some, override aggressive urges [150]. It is also worth noting the human-subject experimental studies showing that the mere provocation of perceived poverty, powerlessness, or the perception of belonging to an underprivileged “out-group” leads to less healthy, energy-dense dietary choices [151,152,153]. While there appear to be no significant changes to mood or behavior among healthy adults in the 160 min following a glucose tolerance test in nonstressful conditions, even as blood levels approached hypoglycemic-like zones [154], it would be interesting to observe whether or not that is the case under conditions of social rejection. Since advances in neuroimaging are aiding in the study of aggressive behavior [155], it would also be useful to pair hypoglycemic-like states with objective psychophysiology and neuroimaging [156,157]. The link between sugar and human behavior is a complex topic, but pre-clinical studies continue to indicate that chronic early-life sugar consumption promotes later-life aggression [158]. It is also possible that the type of sugar source (e.g., sucrose vs. maple syrup) might be an important consideration in the context of the microbiome, a topic to be explored later [159].

## 4. Dietary Intervention Studies

Despite Senator George McGovern’s 1977 call for funding and support for high-quality research directed at nutrition and mental health, such research was not forthcoming. Certainly, volumes of bench studies and animal experiments have shown that lab chow high in fat, sugar, and/or synthetic additives can influence brain function and behavior. Only in recent years, though, have controlled human intervention studies examined dietary patterns and neuropsychiatric outcomes. These studies have generally shown that shifting away from high-sugar, high-fat processed foods and toward more nutritious whole foods can improve neuropsychiatric outcomes, including depression. For example, the SMILES trial (*n* = 67), a 3-month healthy diet intervention, improved ratings of depression on a clinical rating scale compared to a social support control group. The dietary intervention involved personalized dietary recommendations that were not dissimilar to Schoenthaler’s studies—with an emphasis on fruits, vegetables, whole grains, nuts, and dairy without added sugars, with the exclusion or minimization of ‘extras’ foods, such as sweets, refined cereals, fried food, fast food, processed meats, and sugary drinks (no more than 3 per week) [160]. In a shorter, 3-week study with similar dietary recommendations, young adults (*n* = 101) were randomized into a dietary intervention or habitual diet group; the results showed that the diet intervention group had significantly lower self-reported depression symptoms at the end of the study period and at a follow-up 3 months later [161]. In a multi-center, randomized controlled trial (*n* = 292) involving a low-fat, low-glycemic index, plant-based diet intervention, the adults in the diet group reported improved mental outlook and productivity [162]. Other randomized controlled intervention studies with similar designs have demonstrated that improvements in mental health are noted with a greater inclusion of fruits, vegetables, lean meats, fish, whole grains, and the exclusion of highly processed snacks and fast food [163,164,165,166]. The design of these studies often includes instruction from dietetics professionals who advise on food “swaps” [164], which is precisely what Schoenthaler was attempting to do in his whole-of-diet studies [63]. It is likely that the removal of ultra-processed foods is a critical aspect of the positive outcomes observed in the diet–mental health studies [167]. Importantly, researchers have estimated that dietary intervention studies have the potential to save millions of dollars in healthcare costs alone [168,169]. With a further expansion of intervention studies, it might one day be possible to estimate costs in the context of lower recidivism, as claimed by probation officer Reed in the 1970s.

In addition to whole-of-diet studies, there are also recent trials that have focused on select foods, including those high in polyphenols. Plant foods that have not been subjected to ultra-processing are typically rich in phytochemicals that give plants their taste, texture, and color. Among phytochemicals, there are some 8000 polyphenols, a wide range of compounds with antioxidant and anti-inflammatory properties. Dietary polyphenols have been associated with a number of health outcomes, including better mental health [170]. Volumes of animal research show that dietary polyphenols can target the brain, reducing psychological distress in various models [171,172]. Recently, intervention trials that have specifically targeted increases in dietary polyphenols (e.g., the inclusion of red and purple berries, cocoa, or whole fruit juices) have shown improved cognition and overall mental health [173,174,175,176].

At this juncture, it is worth mentioning Schoenthaler’s 1983 orange juice study; in a secure juvenile detention facility in Virginia, the baseline meal-time condition was one in which the youths sat at eight-person tables. Each table held one pitcher of water and one of milk. Schoenthaler’s design added another pitcher to each table. The third pitcher contained 100% orange juice. The youth regulated their own intake of water, milk, and/or orange juice. The overall milk consumption did not change during the study, and the subjects were, on the whole, increasing their consumption of orange juice instead of water. Schoenthaler examined the disciplinary records of the 239 juveniles who were in the facility for the six-month period predating the orange juice exposure. These records were graded against those of the 242 juveniles present during the orange juice exposure. Schoenthaler found a 47% reduction in antisocial behavior per juvenile per day during the juice exposure period [177]. Obviously, the lack of linkage between individual juveniles and their specific consumption of orange juice is a major limitation when interpreting the results. However, Schoenthaler was essentially mocked by critics who claimed that the results would indicate that an increase in simple sugars (via the juice) was effective in curbing antisocial behavior [178]. Contemporary science informs us that compared to drinks with matched glucose/fructose, orange juice limits the post-prandial oxidative stress and inflammation associated with a high-fat meal [179], and Schoenthaler’s orange juice consumers would have been ingesting bioactive phytochemicals with potential brain-modulating activity [180]. In one recent 8-week study involving young adults, the consumption of 100% orange juice improved mental outlook and depressive symptoms vs. an imitation “fruit drink” with similar glucose/fructose content. The only significant difference in the drinks was the flavonoid content. The actual orange juice contained 158 mg of flavonoids per 100 g, while the imitation juice contained only 28 mg per 100g [181]. Indeed, human research indicates that an improvement in mood via flavonoid-rich orange juice may result from changes to the gut microbiome [182], a topic we turn to next.

## 5. Microbiome and Mechanisms

As mentioned earlier, diets high in ultra-processed foods have recently been linked to low-grade systemic inflammation [40]. This is important because human research indicates that elevated markers of low-grade inflammation (e.g., C-reactive protein) are a characteristic finding in people with mental illness and aggressive tendencies [183,184,185,186]. In a recent study involving 686 adults with bipolar disorder (vs. 343 healthy controls), researchers found that the systemic immune–inflammatory index (a combined measurement of neutrophils, lymphocytes, and platelet counts) was higher among people with bipolar disorder who had committed criminal offenses [187]. Similar findings of increased inflammatory immune markers have been reported for adults with schizophrenia who had committed crimes vs. noncriminal patients [188]. Dietary factors (omega-3 fatty acids, for example) can act as signaling molecules throughout the immune system, limiting systemic low-grade inflammation [189,190]. More specifically, dietary factors can limit the production of proinflammatory cytokines (e.g., interleukin-6, interleukin-1 beta, and tumor necrosis factor-alpha) [191,192], immune chemicals that have been implicated in human aggression [193,194,195]. The observation that omega-3 fatty acids (vs. other dietary fats) have differential effects on gut microbiota helped to strengthen the early arguments that the intestinal ecosystem plays an underestimated role in the diet–mental health linkage observations [196,197].

In his now 50-year-old book *Nutrition and Your Mind*, Watson claimed that the intestinal microbiota were involved in abnormal behavior. He argued that a nutrient-dense, fiber-rich diet supplemented with “healthy” bacteria via yogurt or commercial *Lactobacillus acidophilus* products could “restore” the intestinal microflora. Watson’s contentions were clinical observations and not rooted in any available science. It was not until 1986 that researchers demonstrated, using germ-free vs. conventional mouse models, that the gut microbiota can influence brain physiology [198]. Following this, researchers showed that miniscule amounts of orally administered *Campylobacter jejuni* activate the visceral sensory nuclei in the brainstem, causing distress and anxious behavior in animals [199]; *C. jejuni* is a microbe that causes gastritis in humans, but the amounts administered were far below a pathogenic level, and the mechanism of this gut microbe-induced brain activity was determined to be direct gut-to-brain communication via the vagus nerve [200,201,202]. At the same time, other groups were demonstrating that various forms of stress—heat, cold, acoustic, crowding, physical exhaustion, restraint, food deprivation, maternal separation—could disrupt the normal gastrointestinal microbiota in animals, and yet others were showing that the oral administration of beneficial microbes can lower systemic inflammation; collectively, these studies allowed for the presentation of legitimate biophysiological mechanisms to support Watson’s observations [196,197].

The accumulating research linking alterations of the gut microbiome and aggression and/or antisocial behavior remains largely in the pre-clinical domain. For extensive discussion, the reader is referred to elegant reviews that explore the topic in detail [45,203,204]. Briefly, the microbiota–gut–brain axis is thought to operate through several mechanisms. As described, the vagus and spinal nerves carry microbially mediated information to the brain [205], and gut microbes also influence humoral signaling molecules (e.g., cytokines), neuropeptides, and hormonal messengers that otherwise contribute to mood and behavior [206]. Nutritional status is also influenced by gut microbes through the manufacturing of vitamins, the absorption of nutrients, including omega-3 fatty acids [207,208], and by acting upon dietary components ranging from amino acids (e.g., tryptophan, the serotonin precursor) to the aforementioned polyphenols (i.e., producing more bioactive metabolites) [209]. A loss of integrity of the intestinal barrier (i.e., the gut “lining”) through ultra-processed diets and/or psychological stress [210,211] can initiate a cascade of low-grade inflammation and metabolic dysregulation with consequences for mood and aggression [212,213,214]. The consequences of increased intestinal permeability (or so-called “leaky gut”) appear to include increased aggression [213]. Researchers are actively trying to determine whether certain gut microbial signatures are associated with temperament [215], violent tendencies [216], and the regulation of emotions [217]. Emerging human studies using specific strains of probiotics indicate that targeting the gut microbiome might lower aggressive thoughts [218] and impulsivity [219].

As researchers learn more about the ways in which dietary patterns influence the gut microbiome, it is becoming clear that various dietary components may influence the brain and behavior, both positively and negatively, via the microbiome [220]. Dietary patterns high in ultra-processed foods (vs. minimally processed, polyphenol, and fiber-rich diets) are associated with negative alterations to the gut microbiome, which in turn appear to influence metabolism and behavior [221,222,223,224]. Emerging human research shows that dietary interventions directed at the microbiome (e.g., whole grains, onions, leeks, cabbage, oats, fermented foods) can reduce psychological stress and improve mood [225,226]. In animal models, when the fecal microbes from animals reared on Westernized dietary patterns are transferred to otherwise healthy animals consuming normal lab chow, the recipient animals show cognitive deficits and behavioral changes [227,228,229]. Remarkably, a new study currently in preprint shows that fecal microbiome transplants from one-month-old human infants prescribed antibiotics during their first days of life (i.e., in a state of dysbiosis) lead to significant increases in aggression in recipient mice [230]. Chronic unpredictable stress also disturbs the gut microbiome, and the transfer of fecal microbes from animals that experienced chronic unpredictable stress to healthy animals leads to anxiety and depressive-like symptoms among otherwise normal recipient animals [231]. This suggests that the gut microbiome, as influenced by regular dietary intake or chronic stress, plays a significant role in mental outlook and behavior. Obviously, correction facilities are places where chronic, unpredictable stress abounds, which might suggest that targeting the microbiome, as Schoenthaler almost certainly did (albeit inadvertently), is an important consideration.

## 6. Food Additives

At first glance, it might appear that the harmful aspects of ultra-processed foods are a product of their high fat and high sugar content and/or the absence of dietary fiber [232,233]. However, it is becoming increasingly clear that dietary additives are associated, but independent, contributors to chronic disease [234]. In our context of criminal justice, emerging research (described below) indicates that multiple food additives, including emulsifiers, synthetic colors, and flavor enhancers common to ultra-processed foods, can also influence brain and behavior.

In animal models, various dietary additives, including emulsifiers, aspartame, and monosodium glutamate (MSG), have been shown to alter the gut microbiome [235,236,237,238,239,240]. Dietary emulsifiers, such as carboxymethylcellulose and polysorbate-80, increase sensitivity to social stress in animals and can alter gene expression in the amygdala [241,242]. Animal models also demonstrate that early-life exposure to dietary emulsifiers appears to have long-lasting influences on cognitive impairments and the expression of anxiety-like traits [243].

Glutamate (the main component in MSG) and associated “dietary excitotoxin” chemicals such as aspartate have been linked to pain sensitivity and various neuropsychiatric symptoms, possibly because these agents can overexcite neurons [244]; a large number of pre-clinical studies support the idea that MSG can influence pain and lead to abnormal behavior in animals, including those that mimic depression and/or anxiety [245,246,247]. Recent human studies have found that the elimination/low intake of excitotoxin additives, including aspartame and MSG or MSG-like chemicals, can improve symptoms of depression, anxiety, posttraumatic stress disorder (PTSD) [248,249], and fibromyalgia [250]; this includes improved depression, anxiety, and cognitive function and reduced pain sensitivity in veterans with Gulf War Illness [251,252,253]. Remarkably, gut microbes appear to influence blood–brain barrier (BBB) permeability via the expression of tight junction proteins [254], and an ultra-processed dietary pattern, at least in animals, can increase the permeability of both the gut and blood–brain barriers [255]. Moreover, disturbances to normal BBB structure and function can be influenced by psychological trauma and acute and chronic stress and may have a bidirectional relationship with mental illness [256]. Differential levels of intestinal and/or BBB permeability can help explain why subsets of the population might be more vulnerable to the consumption of dietary excitotoxins. It is also interesting to note that among the ultra-processed foods connected to depression, those containing artificial sweeteners appear to have the strongest relationship [30].

While Schoenthaler argued that the removal of dietary additives may have played a role in his results, the idea that dietary additives play a significant role in behavior was largely dismissed as pseudoscience. In particular, the notion that synthetic food colorings could disturb behavior in children was considered to be a fringe concept; however, a tightly controlled study by McCann and colleagues, published in the *Lancet*, demonstrated that synthetic colors and the commonly used artificial preservative benzoate can provoke behavioral changes in otherwise healthy children [257]. Recently, artificial food colors have been linked to changes in brainwave activity and neuropsychiatric symptoms in adults with attention-deficit/hyperactivity disorder (ADHD) [258].

## 7. Food Equality and Prevention

Historically, much of the discourse on nutrition and criminology, or the criminal justice system writ large, has focused on specific macronutrients (e.g., sugar) and select dietary supplements (e.g., multivitamins, omega-3 fatty acids, probiotics). More specifically, the existing research on nutrition and dietary supplements has, understandably, focused on juveniles and adults already in confinement. The landmark 2020 report *Eating Behind Bars*, compiled by Impact Justice, underscores that ultra-processed foods with multiple additives are commonly served in US jails and prisons; most states devote less than USD 3.00 per inmate per day for prison catering, so it is little wonder that technical minimum nutrient requirements are met by the inexpensive fortification of cheap ultra-processed foods. As the report correctly points out, there are inmate behavioral implications vis-à-vis subpar prison food that can extend beyond the facility to post-release [259].

At the same time, there has been less emphasis on prevention. Emerging nutritional psychiatry research is intersecting with a widespread recognition that ultra-processed foods are harmful and that the nutritional playing field is far from level. That is, the access to and affordability of healthy, minimally processed foods is enjoyed by the socioeconomically advantaged at the personal and community levels [260]. How might the larger nutritional landscape influence the risk of entry into the criminal justice system? This should include consideration of so-called ‘obesogenic’ environments where the presence or absence of convenience stores, fast food outlets, supermarkets, grocery stores, full-service restaurants, and fruit/vegetable markets intersects with other factors related to neighborhood safety, including night light, crime rate, traffic volume, and other factors of injury [261]. It can also examine how these neighborhood ‘food’ factors are related to allostatic load and the exposome [97]; the former, allostatic load, refers to the physiological dysregulation associated with chronic-stress-induced stimulation of neuroendocrine, metabolic, autonomic, and immune mediators; the related concept of the exposome refers to the total accumulated environmental exposures (both detrimental and beneficial) that can help predict the biological responses of the “total organism to the total environment” *over time* [262,263].

Research shows that ultra-processed food consumption is higher in adults with lower income, less education, and people living with food insecurity [264]; in the US, fast food consumption is particularly high among young Black adults [265]. On the other hand, fruit, vegetable, and omega-3 intake is lower in people living with socioeconomic disadvantage [266]. We can observe lower blood levels of dietary antioxidants in association with lower socioeconomic positions [267,268]. As we and others have pointed out previously, marginalized and vulnerable populations are contending with a biopsychosocial intersectionality that adds to health risk; affluent persons, even if they are in the same 50–60% ultra-processed food intake range as persons living with poverty, consuming the same average level of fast food, enjoy multiple health advantages, including the coincident consumption of other polyphenol-, fiber-, and omega-3-rich foods [97,269].

While much of the discourse has focused on relationships between ultra-processed food consumption and obesity, to date, we know little about the intersectionality of food systems and criminal justice. Is it merely a coincidence that the 10 states with the highest obesity rates in 2021—Louisiana, Mississippi, Oklahoma, Arkansas, Kentucky, West Virginia, South Dakota, Alabama, Missouri, and Ohio [270]—are all in the upper echelon of states with the highest 2021 incarceration rates (+325 per 100,000)? In fact, Mississippi, Louisiana, Arkansas, and Oklahoma have incarceration rates of +500 per 100,000 people of all ages [271]. How do food-related policies and practices intersect with political environments and behavior [272]? Black people have the highest incarceration rates in the US [271], and given the emerging research on structural racism in food inequalities [260], including the clustering of fast food outlets [273,274,275,276] and targeted marketing of unhealthy foods to vulnerable populations [277,278,279], the topic of nutritional criminology should be more prominent within biopsychosocial discourse.

Discussions of food inequalities and food justice are an important part of the emerging concept known as the commercial determinants of health [280]. Taken together, the findings above suggest that the ultra-processed food industry has so comprehensively insinuated itself into trusted institutions and assumed the historical identity (i.e., the role) of government that it begins to resemble the pervasive problem of identity theft. Through various tactics, the ultra-processed food industry (as well as the tobacco and alcohol industries) displaces the roles of civil society, nonconflicted academia, and government writ large. These tactics include, but are not limited to, i. regulatory capture: wherein self-regulatory codes of conduct are introduced by the industry; ii. discourse capture: wherein the dominant discourse is about personal responsibility (therefore absolving the industry of harm and deflecting from social structures); iii. program capture: wherein the industry provides ‘education’ programs often targeted at adolescents or programs, e.g., physical activity programs which remove the focus on ultra-processed foods; iv. research capture: wherein the industry co-opts researchers and creates alternative streams of pro-industry research; and iv. treatment capture: wherein the industry provides funds for the treatment of harm, but again this takes any focus from the need to prevent the harm [281,282,283]. To the extent that nutrition influences behavior, experts across the criminal justice continuum should closely scrutinize the tactics of the ultra-processed food industry, especially in the context of food insecurity and food and social inequalities [284,285,286,287].

## 8. Conclusions

The development of the NOVA food classification system [288] has led to ease in the identification of foods that meet specific ultra-processed food criteria; historically, terms such as ‘junk food’, ‘highly-processed food’, and ‘convenience food’ have been used interchangeably, but the NOVA system allows for a common language and research efforts with greater specificity [289]. In recent years, there has been growing recognition that higher ultra-processed food consumption is associated with multiple forms of harm, including those related to mental health and behavior. However, to date, there has been little attention given to the implications of such research on the criminal justice system. Of course, it is not our contention that dietary patterns—nutritious and minimally processed or ultra-processed, inclusive of many synthetic additives—are the sole factor in crime-related behaviors or forensic matters. Antisocial behavior is a complex, multifactorial human phenomenon. Based on the science reviewed in this perspective article, we only argue that dietary factors might be more significant than currently appreciated.

We can only speculate as to why the early 1980s research on dietary patterns and aggressive behavior in detention facilities was marginalized and mostly left unattended without replication efforts; it is possible that sensationalized criminal trials, wherein defense attorneys claimed that the accused committed homicide due to sugar or monosodium glutamate-containing foods—the so-called “Twinkie Defense”—set back interest in the field [63]. It is also true that early findings did not have the buttress of strong biophysiological mechanisms. However, we suspect, based on the research reviewed above, that the long arm of industry influence has played a role in minimizing relationships between food and behavior. Only recently have we learned the extent of the ultra-processed food industry’s influence on policy [290,291,292] and nutritional professional groups, such as the Academy of Nutrition and Dietetics (i.e., the group that quickly dismissed a diet–criminal behavior link in 1985) [293,294].

Research in the fields of nutrition and behavioral sciences has advanced substantially since the 1980s. There has been growing interest in the fields of nutritional neuroscience and nutritional psychology/psychiatry, exemplified by the 2023 Continuing Education module on nutrition and mental health published in the American Psychological Association’s *Monitor on Psychology* [295]. However, academic and public discussions of the increasingly robust research on nutrition, brain, and behavior (and the role of ultra-processed foods in that discourse) seem to elide the topic of criminal justice. Of course, the study of ultra-processed foods vis-à-vis criminology is a complex endeavor that will require a closer examination of the ways in which the consumption of such foods is associated with risk-taking behavior, including the use of alcohol and illicit drugs [296,297]. We are hopeful that our writings here will spark further hypotheses and transdisciplinary collaboration. It will also require a closer examination of the tactics used by the purveyors of ultra-processed foods, including attempts to influence academia and engage in public campaigns to obfuscate critical research findings while manufacturing broad perceptions of lack of harm [284,285]. Perhaps it is time to apply advanced research techniques and interdisciplinary approaches to revisiting the study of ultra-processed foods as an influencing factor in criminal behavior.

## Data Availability

No new data were created or analyzed in this study. Data sharing is not applicable to this article.
